# TMPRSS4 Promotes Cell Proliferation and Inhibits Apoptosis in Pancreatic Ductal Adenocarcinoma by Activating ERK1/2 Signaling Pathway

**DOI:** 10.3389/fonc.2021.628353

**Published:** 2021-03-18

**Authors:** Jianyou Gu, Wenjie Huang, Junfeng Zhang, Xianxing Wang, Tian Tao, Ludi Yang, Yao Zheng, Songsong Liu, Jiali Yang, Liwei Zhu, Huaizhi Wang, Yingfang Fan

**Affiliations:** ^1^Department of Hepatobiliary Surgery I, Zhujiang Hospital, Southern Medical University, Guangzhou, China; ^2^Institute of Hepatopancreatobiliary Surgery, Chongqing General Hospital, University of Chinese Academy of Sciences, Chongqing, China

**Keywords:** TMPRSS4, proliferation, apoptosis, ERK1/2, pancreatic ductal adenocarcinoma

## Abstract

Transmembrane protease serine 4 (TMPRSS4) is upregulated in various kinds of human cancers, including pancreatic cancer. However, its biological function in pancreatic ductal adenocarcinoma (PDAC) remains unclear. In the current study, real-time qPCR, immunohistochemical staining, Western blotting, and database (Cancer Genome Atlas and Gene Expression) analysis revealed remarkable overexpression of TMPRSS4 in PDAC tissue as compared to non-tumor tissue. The TMPRSS4 overexpression was associated with poor prognosis of PDAC patients. Moreover, multivariate analysis revealed that TMPRSS4 serves as an independent risk factor in PDAC. We performed gain-and loss-of-function analysis and found that TMPRSS4 promotes cellular proliferation and inhibits apoptosis of PDAC cells both *in vitro* and *in vivo*. Furthermore, we showed that TMPRSS4 might promote cell proliferation and inhibit apoptosis through activating ERK1/2 signaling pathway in pancreatic cancer cells. These findings were validated by using ERK1/2 phosphorylation inhibitor SCH772984 both *in vitro* and *in vivo*. Taken together, this study suggests that TMPRSS4 is a proto-oncogene, which promotes initiation and progression of PDAC by controlling cell proliferation and apoptosis. Our findings indicate that TMPRSS4 could be a promising prognostic biomarker and a therapeutic target for the treatment of pancreatic cancer.

## Introduction

Pancreatic ductal adenocarcinoma (PDAC) is the most common type of pancreatic cancer (PC) and the fourth most lethal malignancy. It has a poor prognosis with the five-year survival rate of only 10% (1). There has not been much progress in the early prognosis and treatment in pancreatic cancer as compared to other malignancies, such as breast and colorectal cancers (2). Clinical management of pancreatic cancer poses several challenges such as prevention, early diagnosis at a curable stage, and effective biomarkers for its precise detection (3). Therefore, there is an unmet need for the development of sensitive, as well as specific therapeutic targets and an in-depth understanding of the regulatory mechanisms that mediate the development of pancreatic cancer.

Transmembrane protease serine 4 (TMPRSS4), previously referred to as TMPRSS3, is a protein-coding gene located in the human chromosome 11q23.3 (4). TMPRSS4 belongs to the type II transmembrane serine protease family. This family of proteins is anchored to the plasma membrane by the transmembrane domain. They play crucial roles in the execution of diverse cellular functions (5, 6). Cancer cells have distinct characteristics of abnormal cell proliferation, apoptosis inhibition, active migration, and invasion. Recent studies suggest that TMPRSS4 facilitates cancerous tumor growth and metastasis in multiple malignancies. A positive correlation between the overexpression of TMPRSS4 and poor prognosis was demonstrated in a variety of human cancers (7–20). In lung cancer, TMPRSS4 promotes cell growth, and imparts drug resistance to chemotherapy (21). In pancreatic cancer TMPRSS4 is overexpressed and involved in metastasis formation and tumor invasion (4).

Additionally, TMPRSS4 enhances cell invasion by activating NF-kB/MMP-9 signaling pathway in human gastric cancer cells (22). Also, TMPRSS4 mediates an epithelial to mesenchymal transition phenotype by the activation of distinct signaling pathways such as FAK, ERK1/2, Akt, Src, and Rac1 (5). Previous studies indicate that TMPRSS4 might play a crucial role in the development and progression of tumors. However, the underlying molecular mechanisms of TMPRSS4 in pancreatic cancer development and progression remain unclear.

In this study, we unraveled that overexpression of TMPRSS4 is associated with poor prognosis in pancreatic cancer. We also found that TMPRSS4 mediates cell proliferation and inhibits apoptosis in pancreatic cancer cells. These findings suggest that TMPRSS4 is involved in the development and progression of pancreatic cancer. Further in-depth investigation of TMPRSS4 might lead to its development as an efficient prognostic biomarker and therapeutic target for pancreatic cancer.

## Materials and Methods

### GEO and TCGA Data Mining and Analysis

We probed the GEO database (https://www.ncbi.nlm.nih.gov/geo/) to explore the expression of TMPRSS4 in PDAC and non-cancerous tissues. We found overexpression of TMPRSS4 in the datasets of GSE62165 (118 PDAC samples and 13 non-tumor samples) (23), GSE15471 (36 paired PDAC tissues) (24, 25), GSE62452 (69 pancreatic tumors and 61 adjacent non-tumors) (26), and GSE16515 (36 pancreatic tumor samples and 16 non-tumor samples) (27). We validated the aberrant upregulation of TMPRSS4 by integrating analysis of the TCGA (The Cancer Genome Atlas) data and GTEx data (https://xenabrowser.net/datapages/) (28). The association between the TMPRSS4 expression and the prognosis of PDAC patients was investigated in the 149 (all 177 pancreatic cancer) and 36 PDAC cases from TCGA and GSE16515, respectively.

### Tissue Specimens

The current study is approved by the Southwest Hospital Ethics Committee. Tissue specimens were procured from the Southwest Hospital, Third Military Medical University in Chongqing, China. We performed immunohistochemical analysis in a tissue microarray containing 97 cases of histopathologically diagnosed PDAC and 12 normal pancreatic tissues. Additionally, 14 fresh-frozen PDAC tissue samples and matched adjacent non-tumor tissues were taken for RT-qPCR and Western blot analysis.

### Cell Lines and Cell Culture

Seven pancreatic cancer cell lines, AsPC-1, BxPC-3, Capan-1, CFPAC-1, PANC-1, SW1990 (Shanghai Institute of Biochemistry and Cell Biology, China), and Hs766t (ATCC, Manassas, VA, USA), and an immortalized human pancreatic ductal epithelial cell lines: HPDE6-C7 (BeNa Culture Collection, China), were used in this study. These cell lines were maintained in the RPMI 1640 medium (Gibco Invitrogen, Grand Island, NY, USA) supplemented with 10% fetal bovine serum (FBS) (Gibco Invitrogen, Grand Island, NY, USA) and 1% antibiotics, in a humidified atmosphere with 5% carbon dioxide (CO_2_) and 95% air at 37°C.

### RNA Extraction and Real-Time PCR

The total RNA was isolated from the PDAC tissue and PDAC cells with the Trizol reagent (Invitrogen, Camarillo, CA, USA) as per the manufacturer's instruction. Furthermore, RNA concentration was evaluated, and stored at −80°C refrigerator to avoid degradation. Prime Script RT reagent Kit (TaKaRa) and SYBR Premix Ex Taq II (TaKaRa) was used to detect the TMPRSS4 mRNA expression and β-actin was used as an internal control. The 2^−ΔΔCT^ method was used to calculate the relative expression of TMPRSS4. The primers used for the TMPRSS4 was (29): Forward: 5′-CCGATGTGTTCAACTGGAAG−3′ and Reverse: 5′-CCCATCCAATGATCCAGAGT-3′.

### Western Blot

Western blot was performed as previously described (30) using antibodies included those targeting TMPRSS4 (1:800; Proteintech, USA), ERK1/2 (1:1,000; Cell Signaling Technology, USA), phospho-ERK1/2 (p-ERK1/2) (1:1,000; Cell Signaling Technology, USA), Bax (1:1,000; Cell Signaling Technology, USA), Bcl-2 (1:1,000; Cell Signaling Technology, USA), cleaved caspase-3 (1:1,000; Cell Signaling Technology, USA), and β-actin (1:5,000; Cell Signaling Technology, USA). The horseradish peroxidase-conjugated antibody was used as the secondary antibody (anti-mouse 1:5,000; Cell Signaling Technology, anti-rabbit; 1:5,000; Cell Signaling Technology). Protein levels were normalized against the endogenous control, β-actin.

### Immunohistochemistry

Immunohistochemistry was performed as per the manufacturer's instruction (Maixin, Fuzhou, China). The staining intensity and percentage of positively stained cells were used as two essential criteria for IHC scoring. The staining intensity was calculated as follows: 0 (no staining), 1 (weak staining), 2 (moderate staining), and 3 (strong staining). The percentage of the positively stained area was calculated as follows: 0 (no staining), 1 (1–10% staining), 2 (10–50% staining), and 3 (more than 50% staining). The staining index (SI) was calculated by multiplying the values of staining intensity and the percentage of positively stained cells. This evaluation method was employed to categorize the tumor samples into low expression (SI ≤ 4) and high expression (SI ≥ 6) groups.

### Establishing the Knockdown and Overexpression of TMPRSS4 Cell Lines

TMPRSS4 knockdown and overexpression AsPC-1 and PANC-1 cell lines were established by lentivirus-mediated transfection. Human TMPRSS4 siRNA and scrambled siRNA were purchased from RiboBio Co. (Guangzhou, China). The transient transfection was performed using Lipofectamine 3000 (Invitrogen) as per the manufacturer's instructions. We cloned the TMPRSS4-targeting short hairpin RNA (shRNA) and TMPRSS4 coding sequence fragments into the pLKD-CMV-G&PR-U6 vector and pRLenti-EF1a-mCherry-P2A-Puro-CMV-3Flag vector (OBiO, Shanghai, China), to generate shTMPRSS4 lentivirus and TMPRSS4 overexpressing lentivirus, respectively. The PDAC cells were infected with concentrated lentivirus as per the manufacturer's instructions. The eGFP-positive or mCherry positive virus-infected cells were selected using puromycin.

### Inhibition of the ERK1/2 Pathway

The AsPC-1 and PANC-1 cells that stably overexpressed TMPRSS4 and the control cells were treated with the specific ERK1/2 phosphorylation inhibitor-SCH772984 (2 mM; CSNpharm, USA) for 24 h. Control cells were incubated with the same volume of dimethyl sulfoxide (DMSO). The efficacy of SCH772984 inhibition was analyzed by Western blot. These treated cells were used for further analysis.

### Cell Proliferation Analysis

The proliferative ability of PDAC cells was determined by the Cell Counting Kit 8 (CCK-8) assay and the 5-Ethynyl-2′-deoxyuridine (EdU) immunofluorescent assay. The CCK-8 and EdU assay were performed as per the manufacturer's instructions (Dojinodo, Shanghai, China) (RiboBio, Guangzhou, China). The CCK-8 assay was performed by seeding the 5,000 stably transfected cells into 96-well plates and adding the 10 μl of Cell Counting Kit-8 solution to each well-followed by incubation for 2 h at 37°C. The OD value was detected at 24, 48, 72, and 96 h after the cells were seeded. The EdU assay was performed by seeding the 5,000 stably transfected cells into 96-well plates, followed by 48 h of incubation with EdU and immunofluorescence staining.

### Apoptosis Assay

Cellular apoptosis was quantified by flow cytometry using an Annexin V-FITC/PI Staining kit as per the manufacturer's instruction (KeyGEN BioTECH, Jiangsu, China). Briefly, 5 × 10^5^ cells were harvested and suspended in 500 μl of binding buffer containing 5 μl annexin V-FITC and 5 μl PI. These cells were incubated for 15 min at room temperature in the dark. The apoptotic cells were detected by the FACSCalibur (BD Biosciences).

### RNA Sequencing Analysis

The total RNA was isolated from the AsPC-1 cells that were stably transfected with the shTMPRSS4 lentivirus using TRIzol. Subsequently, RNA-seq analysis was performed (Genedenovo Co., Guangzhou, China) in three shTMPRSS4-treated cells along with the appropriate control samples using the Illumina HiSeq 2500 (Illumina, USA). The experimental procedure is as follows: Total RNA was extracted using Trizol reagent kit (Invitrogen, Carlsbad, CA, USA) according to the manufacturer's protocol. RNA quality was assessed on an Agilent 2100 Bioanalyzer (Agilent Technologies, Palo Alto, CA, USA) and checked using RNase free agarose gel electrophoresis. After total RNA was extracted, eukaryotic mRNA was enriched by Oligo(dT) beads, while prokaryotic mRNA was enriched by removing rRNA by Ribo-ZeroTM Magnetic Kit (Epicentre, Madison, WI, USA). Then the enriched mRNA was fragmented into short fragments using fragmentation buffer and reverse transcripted into cDNA with random primers. Second-strand cDNA were synthesized by DNA polymerase I, RNase H, dNTP and buffer. Then the cDNA fragments were purified with QiaQuick PCR extraction kit (Qiagen, Venlo, The Netherlands), end repaired, poly (A) added, and ligated to Illumina sequencing adapters. The ligation products were size selected by agarose gel electrophoresis, PCR amplified, and sequenced using Illumina HiSeq2500 by Gene Denovo Biotechnology Co. (Guangzhou, China).

### Animal Experiments

Four to 6 weeks old female BALB/c nude mice were purchased from the Peking University Animal Center (Beijing, China). TMPRSS4 knockdown or overexpressing mouse xenograft tumor models were established. For TMPRSS4 knockdown tumor model, the mice were divided into two groups (*n* = 8 per group). They were subcutaneously inoculated with 5 × 10^6^ AsPC-1/negative control or AsPC-1/TMPRSS4-shRNA cells in the left oxter. The tumor length (L) and width (W) were monitored every week using a digital Vernier caliper. The tumor volume was determined using the equation (L^*^W^2^)/2. Mice were sacrificed 4 weeks post injection, and tumors were excised and weighed. The mice were sacrificed 4 weeks post injection.

The TMPRSS4 overexpressing tumor model were established to explore the association between the TMPRSS4 and ERK1/2 signaling pathways *in vivo*. Two groups of mice (*n* = 8/group) were subcutaneously inoculated with 5 × 10^6^ AsPC-1/ TMPRSS4 or AsPC-1/Vector cells in the left dorsal flank. One week after injection, mice were randomized into four subgroups (*n* = 4 in each group, with mean tumor volumes of 8.74 ± 2.84, 7.48 ± 1.63, 7.38 ± 0.86, and 7.49 ± 3.35 mm^3^ for the TMPRSS4+DMSO, Vector+DMSO, TMPRSS4+SCH772984, and Vector+SCH772984 group, respectively) and treated with (i, ii) DMSO control, (iii, iv) 25 mg/kg SCH772984 per day by intraperitoneal injection for 3 weeks. The tumor growth was monitored, and tumor excision was performed as mentioned previously. This animal study was approved by the Southwest Hospital Ethics Committee.

### Statistical Analysis

The statistical analyses were performed using SPSS statistical software (version 22.0, Chicago, IL, USA). Data were presented as the mean ± standard deviation (SD) of three independent experiments and analyzed with Student's *t*-test. Statistical tests included in this study for data analysis were the Chi-square test, the log-rank test, and the Student's *t*-test. The Cox proportional hazards regression model was employed to distinguish independent prognostic factors. All tests were two-sided, and statistical significance was defined as *p* < 0.05. Figures were generated using the GraphPad Prism software version 8.0.

## Results

### TMPRSS4 Overexpression in PDAC Is Associated With Poor Prognosis

**Figure 1:**
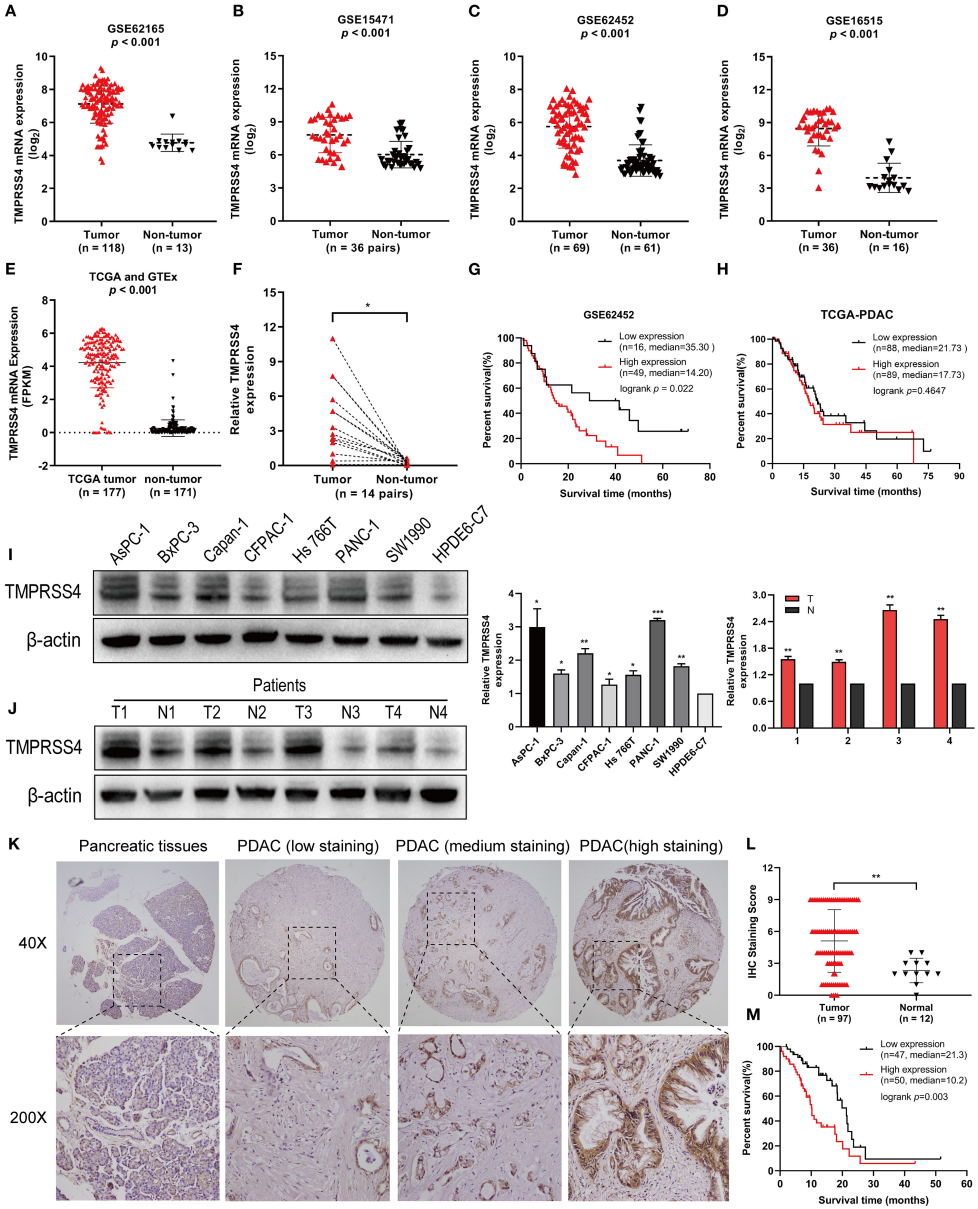
TMPRSS4 is overexpressed in PDAC and associated with poor prognosis. **(A–E)** The mRNAs expression profile showed TMPRSS4 overexpression in pancreatic cancer tissue as compared to non-tumor tissue obtained from the public database (GSE62165, GSE15471, GSE62452, GSE16515, TCGA, and GTEx). **(F)** RT-qPCR analysis on TMPRSS4 in 14 matched PDAC tissues and adjacent non-tumor tissues. β-actin was used as an endogenous control. Paired Student's *t*-test was applied, ^*^P < 0.05. **(G,H)** The Kaplan-Meier analysis depicted the inverse correlation between the TMPRSS4 expression and overall survival in PDAC patients. The available clinical data of PDAC patients were acquired from the GSE62452 dataset (*n* = 65; *P* < 0.05) and the TCGA dataset (149 PDAC; Log-rank test; *P* > 0.05). **(I,J)** Western blot analysis on TMPRSS4 protein expression in seven pancreatic cancer cell lines (AsPC-1, BxPC-3, Capan-1, CFPAC-1, Hs766t, Panc-1, and SW1990) and in the PDAC tissues compared to the primary normal human pancreatic duct epithelial cell (HPDE6-C7) or adjacent non-tumor tissues. β-actin was used as an endogenous control. Data are presented as mean ± SD; ^*^P < 0.05, ^**^P < 0.01, ^***^P < 0.001. **(K,L)** IHC staining demonstrated the TMPRSS4 overexpression in PDAC tissue compared to the pancreatic non-tumor tissue. Data are presented as mean ± SD; ^**^P < 0.01. **(M)** Kaplan-Meier analysis revealed a negative correlation between the TMPRSS4 expression and overall survival of patients (*n* = 97; Log-rank test; *P* = 0.003).

The mRNA expression profiles of the GSE62165, GSE15471, GSE62452, and GSE16515 datasets from the GEO database (https://www.ncbi.nlm.nih.gov/geo/) were analyzed and showed a significant overexpression of TMPRSS4 mRNA in pancreatic cancer tissue as compared to the non-tumor tissue (Figures 1A–D). These findings were validated by data from the Cancer Genome Atlas dataset (TCGA) and the Genotype-Tissue Expression (GTEx) (Figure 1E). Additionally, we performed RT-qPCR to evaluate the TMPRSS4 expression in 14 paired PDAC tissues collected from our hospital. The result demonstrated that TMPRSS4 was upregulated in PDAC tissue, which further confirmed the above data (Figure 1F).

Moreover, analysis of the GSE62452 dataset revealed that TMPRSS4 overexpression is correlated with poor prognosis in pancreatic cancer (Figure 1G). However, in patients with 149 PDAC pathologic type of TCGA dataset pancreatic cancer, Kaplan-Meier analysis showed no significant difference between TMPRSS4 expression and overall survival (Figure 1H). We then further explored the relationship between TMPRSS4 expression and prognosis in all 177 pancreatic cancer patients in the TCGA dataset, and found that they were negatively correlated and TCGA dataset.

In line with these findings, Western blot analysis demonstrated the upregulation of TMPRSS4 in seven pancreatic cancer cell lines (AsPC-1, BxPC-3, Capan-1, CFPAC-1, Hs766t, PANC-1 and SW1990) and in the PDAC tissue in contrast to the primary normal human pancreatic duct epithelial cell (HPDE6-C7) or adjacent non-tumor tissue (Figures 1I,J). IHC staining was followed to investigate the clinical significance of TMPRSS4 expression in PDAC in a tissue microarray containing 97 PDAC tissue and 12 normal pancreatic tissue. TMPRSS4 expression was markedly upregulated in pancreatic cancer tissue as compared to the normal pancreatic tissue (Figures 1K,L). The Kaplan–Meier survival curve and log-rank test demonstrated that TMPRSS4 overexpression was significantly correlated with the poor overall survival in PDAC patients (Figure 1M). We statistically analyzed the IHC score and found that the TMPRSS4 level was strongly correlated with age, tumor size and differentiation (Table 1), We further evaluated the prognostic significance of TMPRSS4 expression by employing univariable and multivariable Cox proportional hazards analysis (Table 2). In univariable analysis, patients with the TMPRSS4 overexpression exhibited the lowest OS (HR, 2.392, *p* = 0.003) (Table 2). We selected the variables whose *p*-value was <0.1 in Cox univariate analysis and put them into Cox multivariate regression model for further analysis. The outcome of our analysis is demonstrated in Table 2, which was in line with our previous analysis (HR, 1.898, *p* = 0.033).

**Table 1:** Clinical characteristics of pancreatic cancer stratified with TMPRSS4 expression.

**Characteristics**	**Low group (*n* = 47)**	**High group (*n* = 50)**	***P*-value**
**Age(years)**			
<60 years	23 (48.9)	13 (26.0)	0.033
≥ 60 years	24 (51.1)	37 (74.0)	
**Gender**			
female	22 (46.8)	16 (32.0)	0.199
male	25 (53.2)	34 (68.0)	
**Tumor size (cm)**			
0–2	7 (14.9)	7 (14.0)	0.021
≥2, <5	38 (80.9)	31 (62.0)	
≥5	2 (4.3)	12 (24.0)	
**Differentiation**			
Moderate-Well	40 (85.1)	31 (62.0)	0.019
Poor	7 (14.9)	19 (38.0)	
**Clinical stage**			
I-II	44 (93.6)	42 (84.0)	0.241
III-IV	3 (6.4)	8 (16.0)	
**Perineuronal invasion**			
No	36 (76.6)	32 (64.0)	0.257
Yes	11 (23.4)	18 (36.0)	

**Table 2:** Cox univariable and multivariable analysis of clinicopathological variables and TMPRSS4 expression in relation to OS in pancreatic cancer patients.

**Clinical factor**	**Univariable analysis**			**Multivariable analysis**		
	**HR**	**95%CI**	***P*-value**	**HR**	**95%CI**	***P*-value**
Age (≥60 vs. <60 years)	1.786	0.984–3.241	0.056			
Gender (male vs. female)	1.649	0.922–2.948	0.092			
Differentiation (Poor vs. Moderate+Well)	2.155	1.163–3.994	0.015	2.353	1.227–4.513	0.01
Tumor size (vs. 0–2 cm)						
≥ 2, <5	2.161	0.664–7.035	0.201	-	-	-
≥ 5	5.133	1.393–18.914	0.014			
Perineuronal invasion (Positive vs. Negative)	1.062	0.565–1.995	0.851			
Clinical stage (III-IV vs. I-II)	6.938	3.051–15.779	<0.001	7.590	3.199–18.009	<0.001
TMPRSS4 expression (High vs. Low)	2.392	1.357–4.215	0.003	1.898	1.052–3.426	0.033

In total TCGA pancreatic cancer, TMPRSS4 expression is associated with prognosis. Probably due to ethnic differences, the expression level of TMPRSS4 was associated with overall survival in this study, which was not in 149 PDAC pathologic type of TCGA. These findings suggest that TMPRSS4 is overexpressed in pancreatic cancer tissue, and it can be an independent prognostic factor in PDAC patients.

### TMPRSS4 Promotes Cell Proliferation and Inhibits Cell Apoptosis in Pancreatic Cancer Both *in vitro* and *in vivo*

The overexpression of TMPRSS4 in multiple malignancies suggests that it could be an oncogenic promoter in PDAC. To explore the biological function of TMPRSS4 in PDAC development and progression, the AsPC-1 and PANC-1 cell lines with TMPRSS4 knockdown and overexpression were established by lentivirus-mediated transfection.

**Figure 2:**
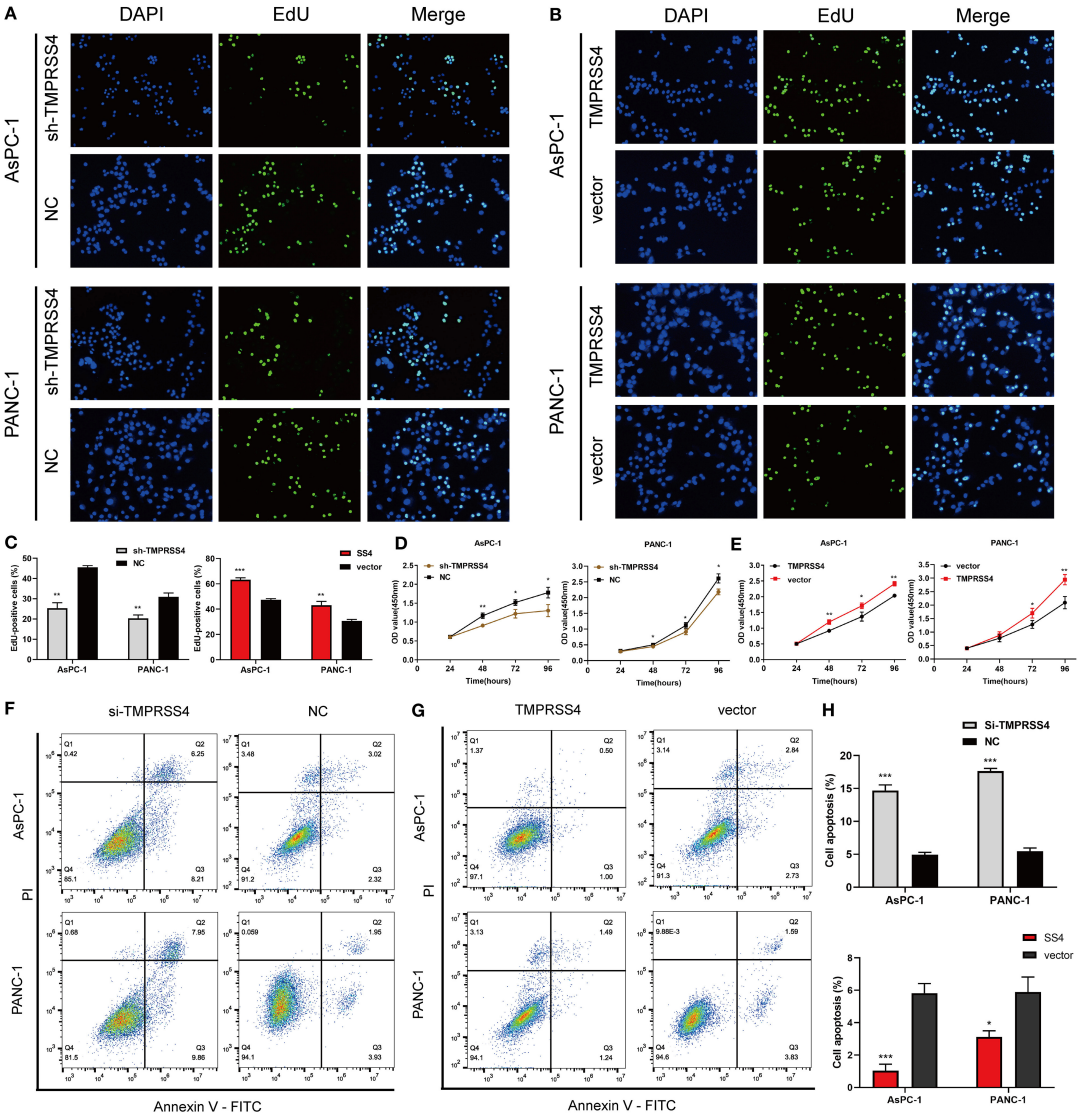
TMPRSS4 promotes pancreatic cancer cell proliferation and inhibits apoptosis *in vitro*. **(A–C)** EdU assays **(A)** decreased cell proliferation in TMPRSS4 knockdown cells; **(B)** enhanced cell proliferation in TMPRSS4 overexpressing cells. (C) Quantification of the EdU staining of indicated cells. **(D,E)** CCK-8 assay on the effect of TMPRSS4 on pancreatic cancer cell growth. **(F–H)** Cellular apoptosis analyzed by flow cytometry. Each bar represents the mean ± SD of three independent experiments. Paired Student's *t*-test was applied. ^*^P < 0.05; ^**^P < 0.01; ^***^P < 0.001; SS4 or TMPRSS4, transmembrane protease serine 4; vector, vector-only control; shTMPRSS4, TMPRSS4 short hairpin RNA; NC, negative control; siTMPRSS4, TMPRSS4 small interfering RNA; PI, Propidium Iodide; FITC, Fluorescein Isothiocyanate.

The EdU and CCK-8 assays were performed and the results indicated a significant decrease in cell proliferation in the pancreatic cancer cells with reduced TMPRSS4 expression (Figures 2A,D). In contrast, cells with induced TMPRSS4 overexpression promoted cellular proliferation (Figures 2B,E). Subsequently, we performed flow cytometry assay to quantify the cellular apoptosis (Figures 2F–H). The percentage of apoptotic cells was significantly higher in siTMPRSS4 pancreatic cancer cells than in the control cells (Figure 2F) but lower in the TMPRSS4 overexpressing cells (Figure 2G).

**Figure 3:**
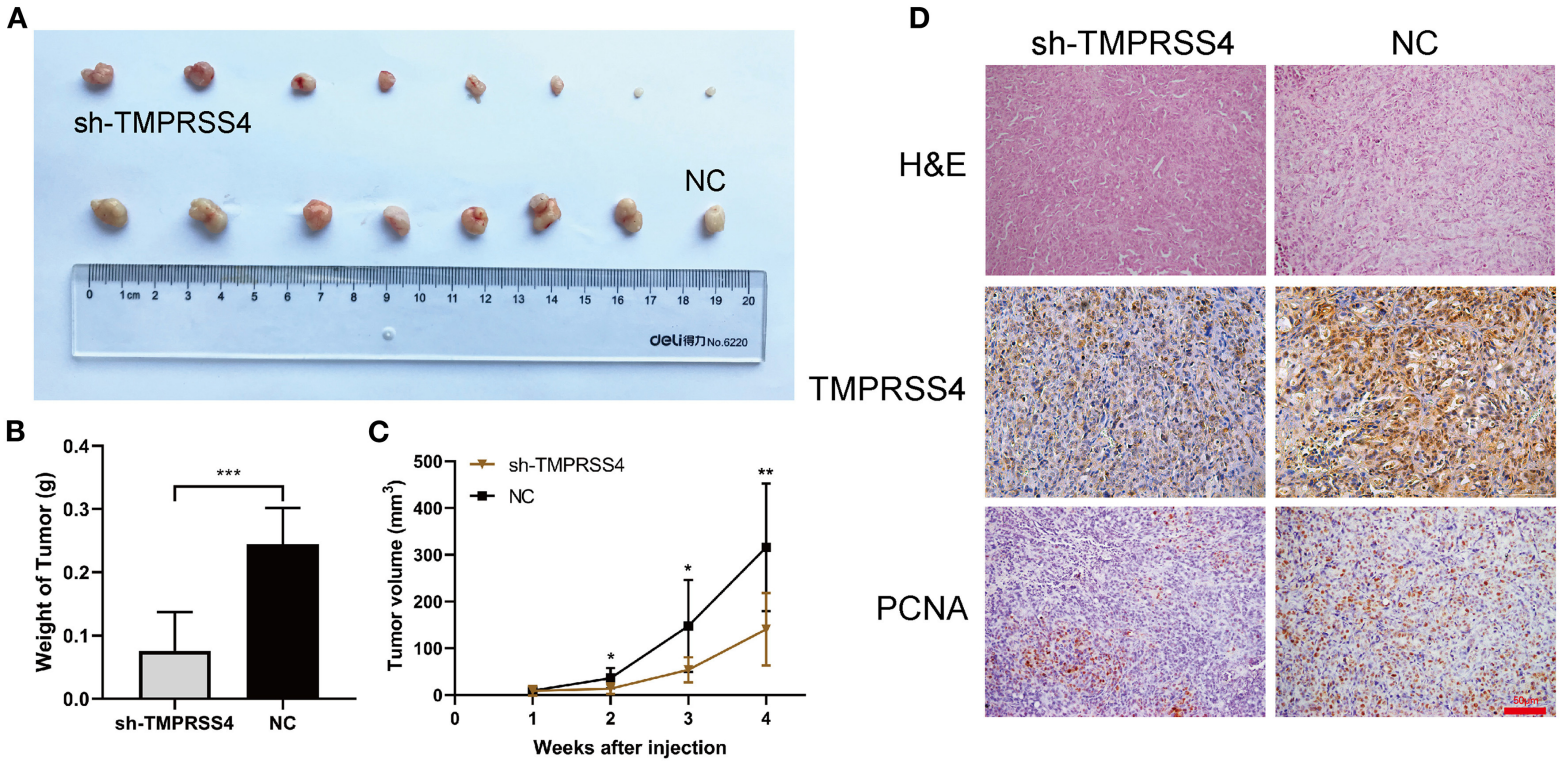
TMPRSS4 promoted the pancreatic cancer cell proliferation *in vivo*. Two groups of BALB/c nude mice were subcutaneously inoculated with 5 ×10^6^ AsPC-1/negative control or AsPC-1/TMPRSS4-shRNA cells. **(A,B)** TMPRSS4 knockdown reduced tumor size as compared to the control group. **(C)** Tumor volumes measured on the indicated days. The tumor length (L) and width (W) were monitored every week using a digital Vernier caliper. And the tumor volume was determined using the equation (L^*^W^2^)/2. Data were presented as the mean ± standard deviation of tumor volume in two groups and analyzed with Student's *t*-test. **(D)** HE and IHC staining on tumor tissues. 200x. Each bar represents the mean ± SD. ^*^P < 0.05; ^**^P < 0.01; ^***^P < 0.001. HE, Hematoxylin and Eosin; IHC, immunohistochemistry; PCNA, Proliferating Cell Nuclear Antigen.

Furthermore, TMPRSS4 downregulation was found to be associated with the reducing tumor volume or weight of the subcutaneous xenografts in nude mice (Figures 3A–C). IHC staining of xenograft tissues showed reduced expression of proliferating cell nuclear antigen (PCNA), a biomarker for cell proliferation (Figure 3D).

**Figure 4:**
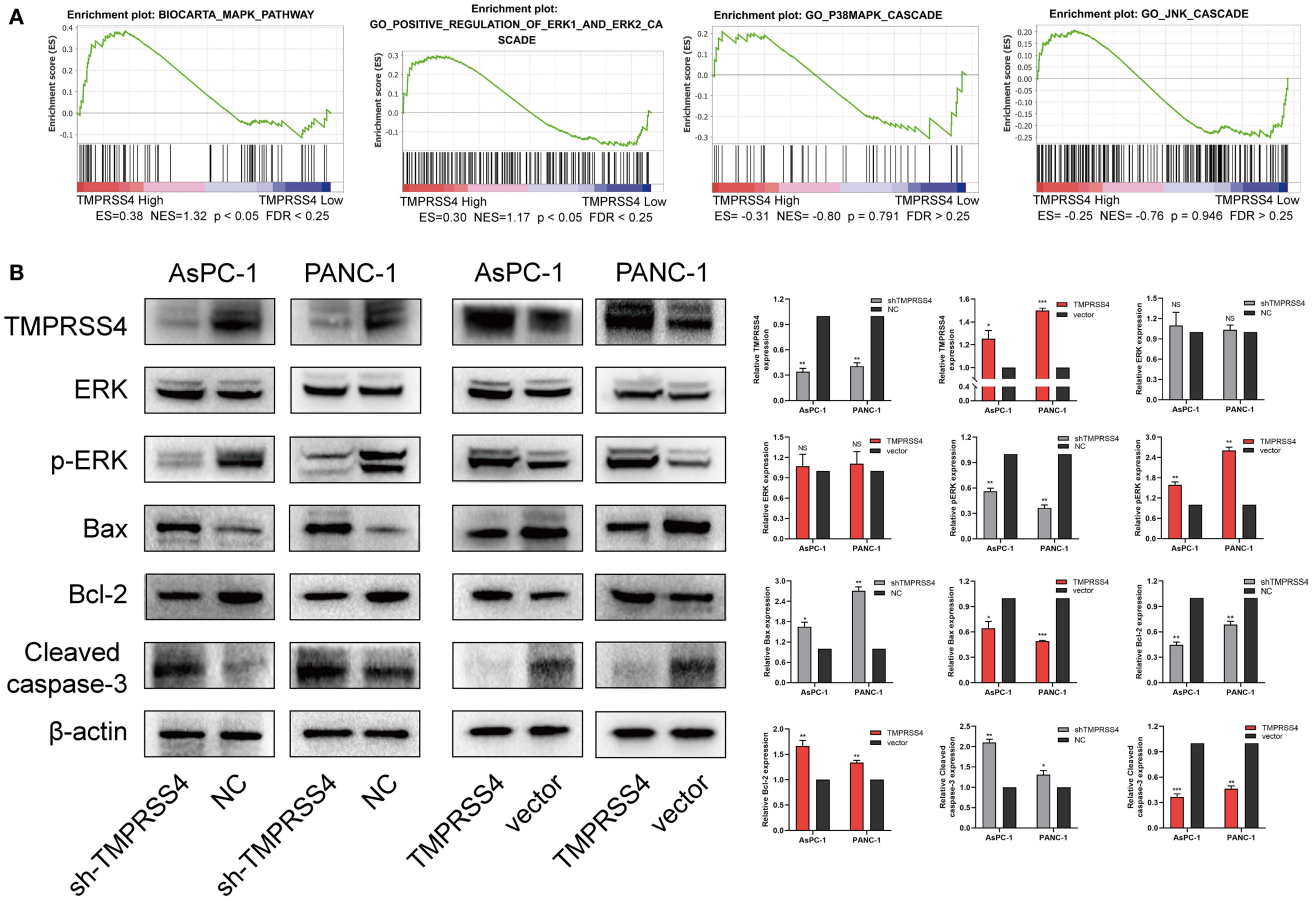
TMPRSS4 activated the ERK1/2 signaling pathway in pancreatic cancer. **(A)** GSEA indicates a significant association between the mRNA expression levels of *TMPRSS4* and the MAPKs pathways, in particular, the ERK1/2 signaling pathway in pancreatic cancer. The other two classical signal pathways of MAPKs, that is, JNK and p38, showed no positive correlation with *TMPRSS4*. **(B)** Western blot analysis of TMPRSS4, total ERK1/2, phosphorylated ERK1/2, Bax, Bcl-2, and cleaved caspase 3; in the indicated cells. β-actin was used as a loading control. Each bar represents the mean ± SD of three independent experiments. Paired Student's t-test was applied. ^*^P < 0.05; ^**^P < 0.01; ^***^P < 0.001; GSEA, Gene Set Enrichment Analysis; MAPKs, Mitogen-activated Protein Kinases.

We also examined the expression of apoptosis-related proteins in the AsPC-1-shTMPRSS4, PANC-1-shTMPRSS4, and control cells (Figure 4B). We found that pro-apoptotic proteins such as Bax, cleaved caspase 3 were increased, and anti-apoptosis protein Bcl-2 was decreased in the TMPRSS4 silencing cells. Conversely, overexpression of TMPRSS4 led to the reduced pro-apoptotic proteins and increased anti-apoptotic proteins. These data indicate that overexpression of TMPRSS4 inhibited apoptosis in the PDAC cells.

Furthermore, TMPRSS4 downregulation was found to be associated with the reducing tumor volume or weight of the subcutaneous xenografts in nude mice (Figures 3A–C). IHC staining of xenograft tissues showed reduced expression of proliferating cell nuclear antigen (PCNA), a biomarker for cell proliferation (Figure 3D). Conversely, upregulation of TMPRSS4 demonstrated opposite effects (Figures 6B–E, SS4+DMSO vs. vector+DMSO). These results suggest that TMPRSS4 plays critical roles in regulating cellular proliferation and apoptosis in PDAC cells.

### TMPRSS4 Activates ERK1/2 Signaling Pathway in Pancreatic Cancer

We performed RNA-seq to investigate which signaling pathways are associated with the TMPRSS4 expression level. Interestingly, the Gene Set Enrichment Analysis (GSEA) revealed that the TMPRSS4 overexpression is strongly correlated with genes associated with Mitogen-activated protein kinases (MAPKs) signaling pathways (Figure 4A). Moreover, previous studies have validated the fact that the activation of ERK1/2 axis contributes to the tumorigenic effect of TMPRSS4 (29, 31). Therefore, we probed the correlation between the TMPRSS4 and the three classical MAPKs signaling pathways, that is, ERK1/2, JNK, and p38 MAPKs signaling pathways, and found that only the ERK1/2 pathway had a positive correlation with the TMPRSS4 expression (Figure 4A). The phosphorylation of ERK1/2 was decreased in TMPRSS4 knockdown cells; whereas it was elevated in TMPRSS4 overexpressing cells (Figure 4B).

### The ERK1/2 Signaling Pathway Is Essential for the TMPRSS4-Induced Proliferative and Apoptotic Behavior in PDAC

**Figure 5:**
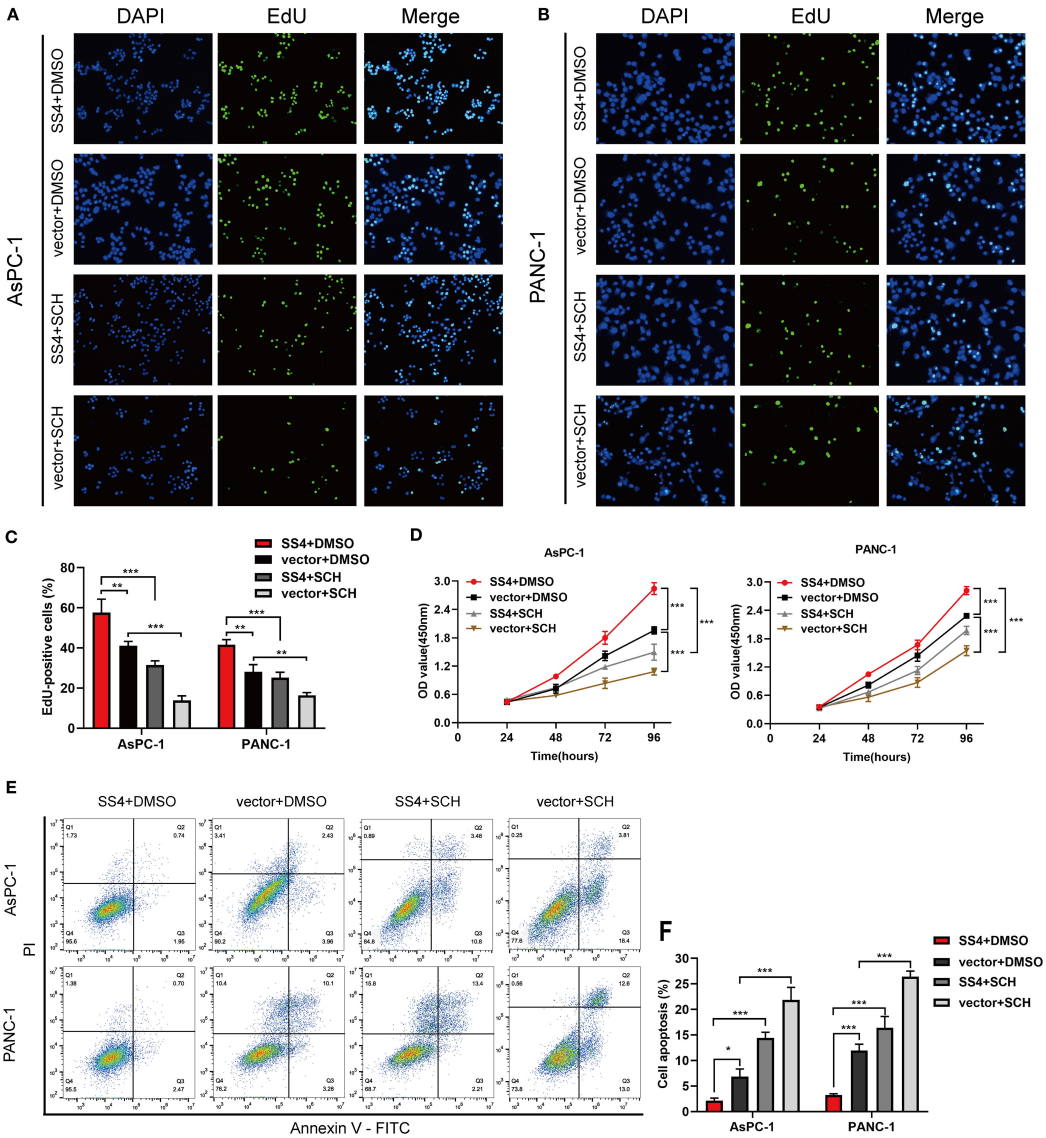
The ERK1/2 signaling pathway is essential for TMPRSS4-induced proliferative and apoptotic behavior of cells *in vitro*. AsPC-1 and PANC-1 cells with stable TMPRSS4 overexpression and the control cells treated with the specific ERK1/2 phosphorylation inhibitor: SCH772984 (2 mM), or the same volume of DMSO for 24 h. **(A–C)** The representative fluorescent micrograph and quantification of the EdU staining of indicated cells. **(D)** The CCK-8 growth curves of indicated cells. **(E,F)** Cell apoptosis analyzed by flow cytometry. Each bar represents the mean ± SD of three independent experiments. Student's *t*-test (for two groups) or ANOVA with Turkey's method (for several groups) were used for comparisons. Paired Student's *t*-test was applied. ^*^P < 0.05; ^**^P < 0.01; ^***^P < 0.001; SS4, transmembrane protease serine 4; SCH, SCH772984; vector, vector-only control; DMSO, Dimethyl Sulfoxide; PI, Propidium Iodide; FITC, Fluorescein Isothiocyanate.

**Figure 6:**
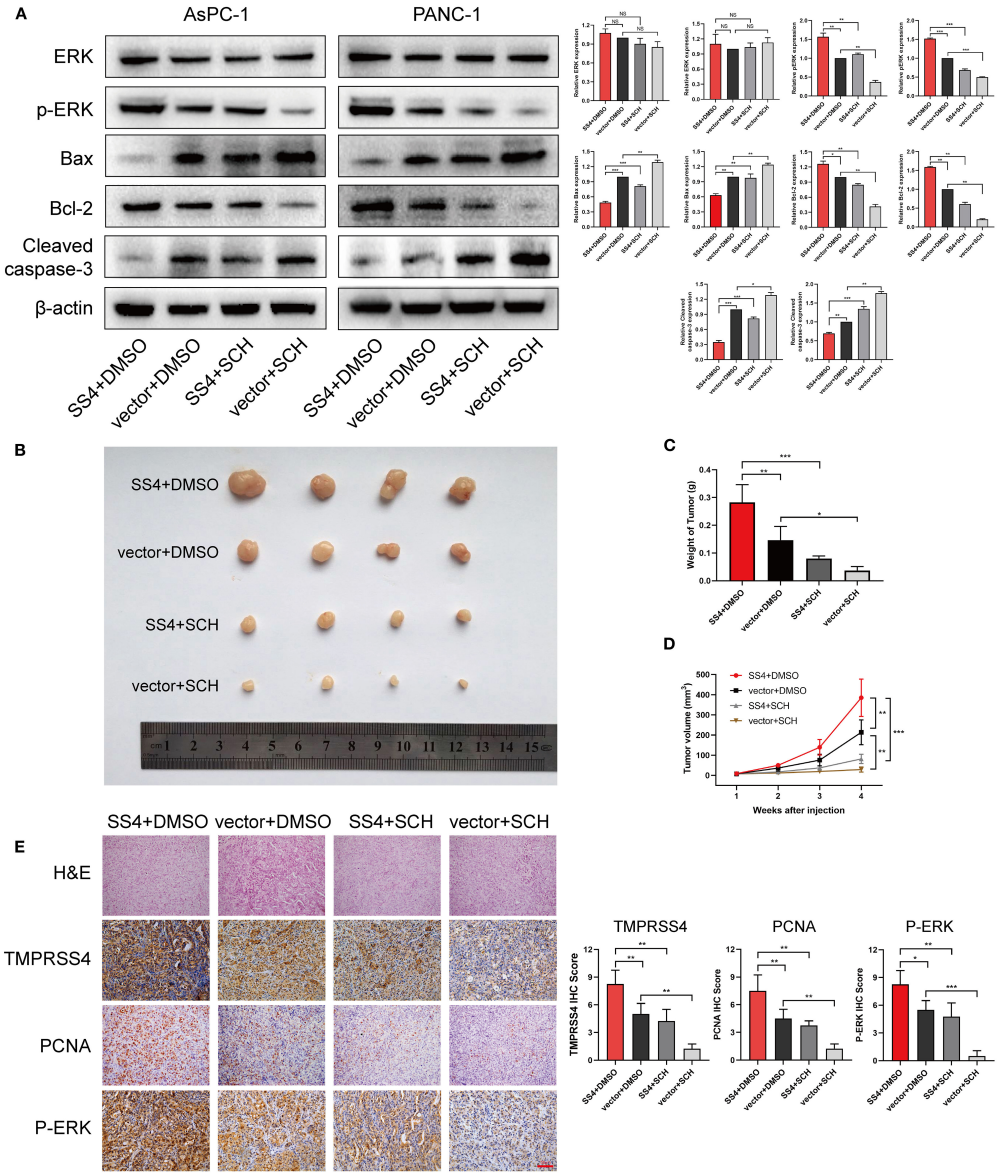
The ERK1/2 signaling pathway is essential for the TMPRSS4-induced cell proliferation *in vivo*. **(A)** Western blot analysis of total ERK1/2, phosphorylated ERK1/2, Bax, Bcl-2, and cleaved caspase 3 in the indicated cells. β-actin was used as a loading control. **(B)** Tumors from four groups of mice. **(C)** The mean tumor weights. **(D)** Tumor volumes measured on the indicated days. **(E)** HE and IHC staining on tumor tissues. 200 x. Each bar represents the mean ± SD. Paired Student's *t*-test was applied. Student's *t*-test (for two groups) or ANOVA with Turkey's method (for several groups) were used for comparisons. ^*^P < 0.05; ^**^P < 0.01; HE, Hematoxylin and Eosin; IHC, immunohistochemistry; PCNA, Proliferating Cell Nuclear Antigen.

We treated the TMPRSS4-overexpressing cells with the ERK1/2 phosphorylation inhibitor, that is, SCH772984, to evaluate the effect of ERK1/2 on the TMPRSS4-induced cell proliferation and apoptosis inhibition. As depicted in Figure 5, the TMPRSS4-induced proliferative and anti-apoptotic behavior was reversed by the SCH772984 treatment. After blocking the ERK1/2 phosphorylation, pro-apoptotic proteins Bax, and cleaved caspase three levels were increased whereas anti-apoptotic protein Bcl-2 level was diminished in the TMPRSS4-overexpressing PDAC cells (Figure 6A). In human PDAC xenograft AsPC-1 models, oncogenic effect induced by the TMPRSS4 overexpression was remarkably inhibited by SCH772984 treatment. In line with the EdU and CCK-8 assays results *in vitro*, IHC staining of the xenograft tumor tissue demonstrated that the PCNA expression was significantly decreased in the SCH772984 treated xenografts (Figure 6E, SS4+DMSO vs. SS4+SCH). Taken together, these results further validate the fact that the activation of the ERK1/2 signaling pathway affects the functional outcome of TMPRSS4, that is, promotes cell proliferation and inhibits apoptosis in pancreatic cancer.

## Discussion

In the present study, we found that TMPRSS4 is significantly overexpressed in PDAC tissues and cell lines. Moreover, overexpression of TMPRSS4 is positively correlated with tumor size and differentiation, also a reduced overall survival rate. Additionally, we performed gain-and loss-of-function analysis and found that TMPRSS4 promotes cellular proliferation and inhibits apoptosis in PDAC. Importantly, the outcome of this study revealed the critical role of TMPRSS4 in mediating cell proliferation and apoptosis in pancreatic cancer cells *via* the ERK1/2 signaling pathway.

TMPRSS4 was found to be overexpressed in multiple human malignancies such as thyroid, lung, breast, pancreatic, gastric, colon, and other cancers. As an oncogenic protein, TMPRSS4 significantly contributes to the development of cancerous tumors. A recent study suggests that TMPRSS4 regulates both proliferation and invasion through Slug and cyclin D1 in prostate cancer cells (31). Similarly, TMPRSS4 facilitates cellular proliferation *via* CREB phosphorylation in thyroid cancer (32). Additionally, TMPRSS4 plays an influential role in radiosensitivity and chemosensitivity by disrupting cell cycle and apoptosis in lung cancer and triple-negative breast cancer (21, 33). These results are in line with our findings that TMPRSS4 accelerates cell proliferation and inhibits apoptosis in PDAC.

Cancer cells are characterized by sustained cell proliferation and diminished apoptosis (34). In the present study, we investigated the role of TMPRSS4 in cell proliferation and apoptosis in PDAC cells. Intriguingly, TMPRSS4 might activate the MAPKs signaling pathways to promote cellular proliferation and suppress apoptosis of PDAC cells.

MAPKs signaling pathways play crucial roles in multiple biological processes by converting the extracellular stimuli into cellular responses. These pathways were reported to be overactivated in human cancers. The MAPKs signaling kinases are chiefly divided into three families: extracellular signal-regulated kinase (ERK), Jun N-terminal kinase (JNK), and p38 MAPKs (35, 36). Previous reports suggest that TMPRSS4 might assist the cellular proliferation, progression, and invasion in lung and colon cancer cells by activating the ERK1/2 and p38 MAPK signaling pathways (29). Similarly, Yunhee Lee et al., reported that the TMPRSS4 induced AP-1 activation is mediated by Axl overexpression and subsequent ERK1/2 and JNK signaling pathway activation, which in turn promotes prostate cancer cell proliferation and invasion (31). Interestingly, in line with these findings, our GSEA results also showed that a higher TMPRSS4 level correlated with gene signatures associated with MAPKs signaling pathways. Further in-depth analysis revealed that among the three classical MAPKs signaling pathways, the ERK1/2 signaling pathway was positively correlated with TMPRSS4 expression, but not JNK and p38 MAPKs signaling pathways.

In the results of genes differentially expressed by RNA-seq, we found MAPKs signaling pathway-related genes such as ACNG4, JUND, TGFA, PTK2B, etc (The annotated informations are in Supplementary Material 3). The bioinformatics analysis revealed that the functions of TMPRSS4 are mediated *via* the ERK1/2 signaling pathway. Our results demonstrated that TMPRSS4 knocked down leads to decreased p-ERK1/2 expression, while TMPRSS4 overexpression leads to increased p-ERK1/2 expression in AsPC-1 and PANC-1 cells. We found that the apoptosis-related proteins Bax, and cleaved caspase 3 were overexpressed while the apoptosis-inhibited protein Bcl2 was downregulated in TMPRSS4 knocked down AsPC-1 and PANC-1 cells. Moreover, we found a decreased expression of Bax and cleaved caspase3 and an increased expression of Bcl2 in TMPRSS4 overexpressing cells.

Caspase 3, a crucial component of the caspase cascade, is activated in both the extrinsic and intrinsic death signal pathways (37). Bcl2 family proteins are key regulators of apoptosis and encompass both proapoptotic proteins (Bid, Bak, Bax, and Bim) and anti-apoptotic proteins (Bcl-2, Bcl-XL, and Mcl-1) (38).

The TMPRSS4 overexpression activates the ERK1/2 signaling pathway and facilitated the subsequent cell survival by upregulating anti-apoptotic protein, Bcl2 and downregulating apoptotic proteins, Bax and cleaved caspase 3. Previous studies have shown that ERK1/2 plays a crucial role in cell apoptosis. Adrian Achuthan et al. reported that suppression of ERK1/2 activity resulted in p90 ribosomal-S6 kinase deactivation and the Bad dephosphorylation, which in turn promoted caspase-3 activity and the subsequent cell apoptosis (39). Another study stated that ACY-1215, an HDAC6-selective inhibitor, inhibited cellular proliferation, and promoted apoptosis *via* PI3K/AKT/mTOR and ERK pathways (40). In this study, we validated that the ERK1/2 signaling pathway is responsible for the TMPRSS4-induced cell proliferative and apoptotic behaviors, that were reversed by ERK inhibitor SCH772984 treatment.

In conclusion, TMPRSS4 is overexpressed in PDAC tissues and cell lines. TMPRSS4, as an oncogene in PDAC, promotes cellular proliferation and inhibits apoptosis by activating the ERK1/2 pathway. Our results suggest that TMPRSS4 protein could serve as a potential biomarker in PDAC prognosis and a potential therapeutic target for PDAC treatment.

## Data Availability Statement

Publicly available datasets were analyzed in this study. This data can be found at: https://www.ncbi.nlm.nih.gov/geo/(GEO database); https://xenabrowser.net/datapages/(TCGA data); https://xenabrowser.net/datapages/(GTEx data).

## Ethics Statement

The studies involving human participants were reviewed and approved by Southwest Hospital Ethics Committee. The patients/participants provided their written informed consent to participate in this study. The animal study was reviewed and approved by Southwest Hospital Ethics Committee.

## Author Contributions

JG and WH contributed to the conception, design, acquisition of data and data analysis, obtaining the findings, and drafting the manuscript. JZ, XW, TT, LY, YZ, SL, JY, and LZ contributed to acquisition of data and technical support. YF and HW offered conception, design and critical revision of the manuscript for important intellectual content. All authors read and approved the final manuscript.

## Conflict of Interest

The authors declare that the research was conducted in the absence of any commercial or financial relationships that could be construed as a potential conflict of interest.
