# Supplementary material for: TMPRSS4 Promotes Cell Proliferation and Inhibits Apoptosis in Pancreatic Ductal Adenocarcinoma by Activating ERK1/2 Signaling Pathway
Source: Front Oncol. 2021 Mar 18;11:628353. doi: 10.3389/fonc.2021.628353 (PMC8012900; doi:10.3389/fonc.2021.628353)
Supplement: Supplementary file 4 [file Data_Sheet_1.docx]

基因信息

TMPRSS4 [Human]

Gene Type: protein-coding

GeneID: 56649

Official Symbol: TMPRSS4

Full Name: transmembrane serine protease 4

Other Aliases: CAPH2 | MT-SP2 | TMPRSS3

>NM_019894.4|TMPRSS4[Human]|CDS 1314bp

atgttacaggatcctgacagtgatcaacctctgaacagcctcgatgtcaaacccctgcgcaaaccccgtatccccatggagaccttcagaaaggtggggatccccatcatcatagcactactgagcctggcgagtatcatcattgtggttgtcctcatcaaggtgattctggataaatactacttcctctgcgggcagcctctccacttcatcccgaggaagcagctgtgtgacggagagctggactgtcccttgggggaggacgaggagcactgtgtcaagagcttccccgaagggcctgcagtggcagtccgcctctccaaggaccgatccacactgcaggtgctggactcggccacagggaactggttctctgcctgtttcgacaacttcacagaagctctcgctgagacagcctgtaggcagatgggctacagcagcaaacccactttcagagctgtggagattggcccagaccaggatctggatgttgttgaaatcacagaaaacagccaggagcttcgcatgcggaactcaagtgggccctgtctctcaggctccctggtctccctgcactgtcttgcctgtgggaagagcctgaagaccccccgtgtggtgggtgtggaggaggcctctgtggattcttggccttggcaggtcagcatccagtacgacaaacagcacgtctgtggagggagcatcctggacccccactgggtcctcacggcagcccactgcttcaggaaacataccgatgtgttcaactggaaggtgcgggcaggctcagacaaactgggcagcttcccatccctggctgtggccaagatcatcatcattgaattcaaccccatgtaccccaaagacaatgacatcgccctcatgaagctgcagttcccactcactttctcaggcacagtcaggcccatctgtctgcccttctttgatgaggagctcactccagccaccccactctggatcattggatggggctttacgaagcagaatggagggaagatgtctgacatactgctgcaggcgtcagtccaggtcattgacagcacacggtgcaatgcagacgatgcgtaccagggggaagtcaccgagaagatgatgtgtgcaggcatcccggaagggggtgtggacacctgccagggtgacagtggtgggcccctgatgtaccaatctgaccagtggcatgtggtgggcatcgttagttggggctatggctgcgggggcccgagcaccccaggagtatacaccaaggtctcagcctatctcaactggatctacaatgtctggaaggctgagctgtaa

Y8261  TMPRSS4 NM_019894 AGGTCAGCATCCAGTACGA

靶点已经在序列中标注出来；
