# Supplementary figures and images for: TMPRSS4 Promotes Cell Proliferation and Inhibits Apoptosis in Pancreatic Ductal Adenocarcinoma by Activating ERK1/2 Signaling Pathway
Source: Front Oncol. 2021 Mar 18;11:628353. doi: 10.3389/fonc.2021.628353 (PMC8012900; doi:10.3389/fonc.2021.628353)

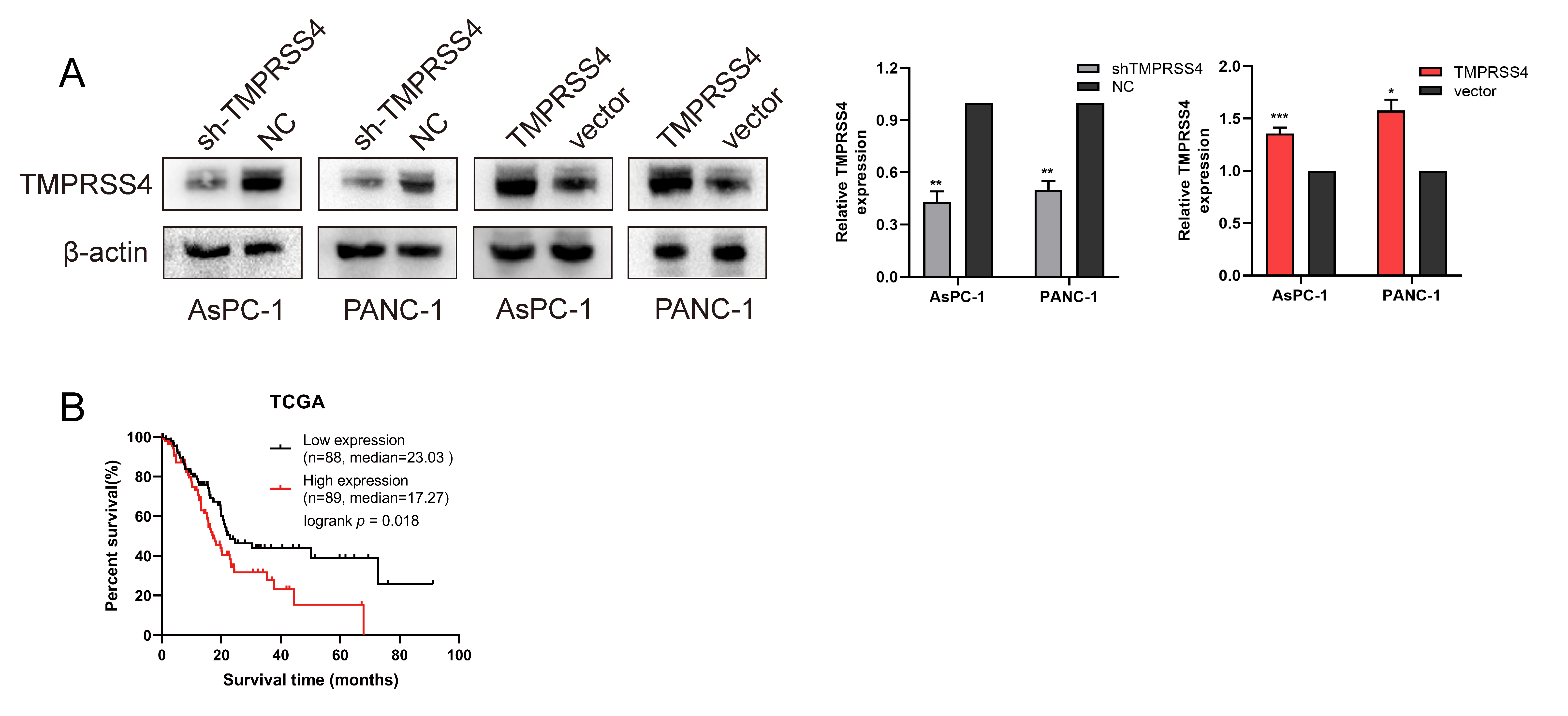

Supplement: Supplementary file 5 [file Image_1.JPEG]
